# Opposite effects of dissolved oxygen on the removal of As(III) and As(V) by carbonate structural Fe(II)

**DOI:** 10.1038/s41598-017-17108-4

**Published:** 2017-12-05

**Authors:** Zeyuan Tian, Yong Feng, Yiyi Guan, Binbin Shao, Yalei Zhang, Deli Wu

**Affiliations:** 1 State Key Laboratory of Pollution Control and Resources Reuse, College of Environmental Science & Engineering, Tongji University, Shanghai, 200092 P.R. China; 2 Department of Civil Engineering, The University of Hong Kong, Pokfulam Road, Hong Kong, China

## Abstract

Freshly prepared carbonate structural Fe(II) (CSF) was used to immobilize As(III) and As(V) in wastewater under oxic and anoxic conditions. Dissolved oxygen was found to exert opposite effects on these two arsenic species. The sorption density of As(III) was higher under oxic conditions, whereas that of As(V) was higher under anoxic conditions. X-ray diffraction and infrared spectroscopic analyses indicated that crystalline parasymplesite (Fe(II)_3_(AsO_4_)_2_·8H_2_O) was formed when As(V) was removed under anoxic conditions, while an amorphous Fe-As-containing precipitate was formed when As(III) was removed under oxic conditions. The distribution of arsenic and iron between the solution and sediments suggested that the oxidation of structural Fe(II) promoted coprecipitation process and inhibited surface complexation. X-ray photoelectron spectroscopic analyses revealed that more As(III) was oxidized under oxic condition, which contributed to a higher sorption capacity for As(III). The formation of parasymplesite through surface complexation/precipitation was proposed to be more effective for the removal of As(V) by CSF, while As(III) was more efficiently removed through coprecipitation. Together, the results suggest that CSF may be an effective material for sequestering both As(III) and As(V). In addition, attention should be paid to the dissolved oxygen content when remediating different arsenic species.

## Introduction

Due to natural and anthropogenic activities, arsenic in surface and ground water has become a hazard to the environment and human health. Exposure to arsenic by ingestion and inhalation may cause acute poisoning and long term lesions, such as cancers of the brain, lung, kidney and bladder^1^. Iron-based substances have been widely used to remove arsenic from aqueous solutions because of the unique affinity of iron to arsenic. Among the various parameters, the dissolved oxygen content has a strong influence on the performance of iron-based adsorbents, and this influence can either be positive or negative. Oxygen has been reported to enhance the removal of arsenic by Fe(II)-containing adsorbents. The adsorption capacity of arsenate (As(V)) and arsenite (As(III)) was higher under aerobic conditions than under anaerobic conditions when synthesized siderite was used as an adsorbent^2^. The removal efficiency of As(III) and As(V) by structural ferrous hydroxide increased from 60% and 50%, respectively, to 90% under aerobic conditions^3^. However, some studies have suggested that limited aeration or even anoxic conditions favour the removal of arsenic. Limited aeration conditions were found to improve the sequestration of As(V) and As(III) by zero valent iron (ZVI)^4,5^. Simon *et al*.^6^ also found that a non-aerated system consisting of a ZVI/sand column had a higher arsenic trapping capacity throughout the reaction period. In another study, the removal capacity of oxidic-shell-free nanoscale ZVI was two times higher under anoxic conditions than in an oxic environment^7^.

The conflicting influence of dissolved oxygen (O_2_) may be due to the impact of O_2_ on the compositions of the iron and arsenic species. In the presence of O_2_, Fe(0) can be oxidized to Fe(II), which is subsequently oxidized to Fe(III). Fe(III) can then undergo hydrolysis and transform into different iron oxyhydroxide and oxide species. The products usually consist of ferrihydrite, green rusts, lepidocrocite, magnetite and goethite^8–10^. Those newly formed oxidative products have been reported to be more active and have higher adsorption capacities than the original iron metal^11,12^. However, continued oxidation would produce a thicker or denser iron oxide film, which may inhibit further oxidation of Fe(0) and consequently deteriorate the performance of the ZVI^13,14^. The mechanisms of arsenic removal vary among the different iron species. Two types of interactions typically occur between Fe-based minerals and arsenic, adsorption and coprecipitation^15,16^. The mechanism of coprecipitation can be further divided into surface complexation and surface precipitation. Numerous studies have claimed that complexation is the predominant bonding mechanism for the adsorption of arsenic onto magnetite, haematite, ferrihydrite, goethite and lepidocrocite^17–19^, while under certain reaction conditions, the removal mechanism involves both complexation and precipitation. Tokoro *et al*.^20^ found that a ferric arsenate and surface complex was formed when As(V) was adsorbed on ferrihydrite with an initial As/Fe ratio above 0.4. Jiang *et al*.^21^ found that the adsorption of As(V) onto ferrihydrite occurred mainly via surface complexation and surface precipitation at acidic pH values (3.0–6.0). The three-dimensional uptake of arsenic by surface precipitation was proposed to have a higher removal efficiency than that of the two-dimensional adsorption of arsenic onto the surface of an adsorbent^20^.

In addition, O_2_ may facilitate the oxidation of As(III) and inhibit the reduction of both As(III) and As(V). The coexistence of iron and O_2_ produces reactive oxygen species, such as ·OH, H_2_O_2_, and O_2_·-^22,23^, as well as Fe(IV)^24^, which can promote the oxidation of As(III). The oxidation of As(III) may lead to an enhanced removal efficiency by adsorbents that have a high affinity for As(V). For those adsorbents that immobilize arsenic by forming solid As(0), such as ZVI, anoxic conditions are beneficial because they preserve the reducing ability of the material^7^. Although a few studies have investigated the effect of O_2_ on the removal of arsenic using Fe-based materials, the effects of O_2_ on the removal of As(V) and As(III) was rarely investigated at the same time. In addition, the roles of the different iron species present during the mineral transformation of iron minerals in the presence of O_2_ need to be further elaborated.

Structural Fe(II) was previously demonstrated to have a high removal capacity towards arsenic^25,26^. In the current study, carbonate structural Fe(II) (CSF) was freshly prepared by a facile coprecipitation method to remove arsenic from water. The objectives of this paper were to (1) determine the influence of O_2_ on the removal efficiency of As(V) and As(III) by CSF; (2) clarify how O_2_ alters the removal mechanism of As(V) and As(III); and (3) provide an environmentally friendly material and supplementary measures to realize the effective remediation of arsenic from water. To understand the roles of the various iron species, the concentrations of ferrous iron and total iron in solution and the sediments were measured. The bonding mechanism was examined through determination of the arsenic species and their distribution in the aqueous and solid phases. The mineral composition of the adsorbents was determined by X-ray diffraction (XRD) analysis and Fourier transform infrared spectroscopy (FTIR). In addition, morphological analysis of the solids was accomplished by scanning electron microscopy (SEM) and transmission electron microscopy (TEM) coupled with energy-dispersive X-ray analysis. The contribution of the redox transformations of arsenic was evaluated by X-ray photoelectron spectroscopy (XPS) analysis.

## Results and Discussion

### Removal of As(III) and As(V) by CSF

**Figure 1:**
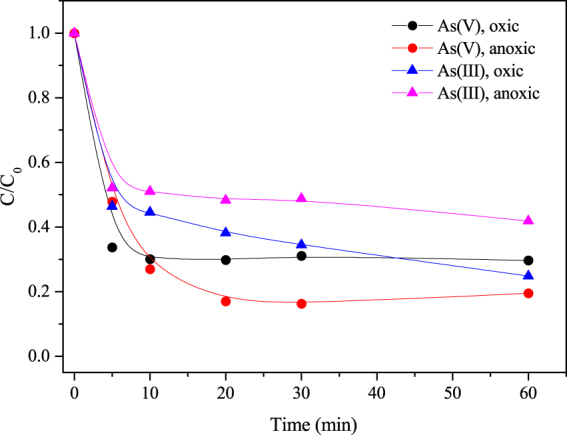
Effect of dissolved oxygen on the removal of arsenic by CSF (As(V) = 100 mg/L, As(III) = 50 mg/L, CSF = 0.1 g-Fe/L, initial pH = 9.0).

Figure 1 shows the oxygen had opposite effects on the removal of As(III) and As(V) at pH 9.0. In general, arsenite is more mobile and more difficult to remove from water than arsenate^27^. Thus the different initial concentrations of As(III) and As(V) was used due to the different removal capacity of CSF towards As(III) and As(V). For a CSF dosage of 0.1g-Fe/L, the removal efficiency of As(V) increased from 70.3% to 80.5% when O_2_ was eliminated and that of As(III) increased from 58.2% to 75.2% when O_2_ was added. The result indicated dissolved oxygen promoted the sequestration of As(III) and inhibited the removal of As(V) by CSF.

### Arsenic and iron distributions in the aqueous and solid phases

**Figure 2:**
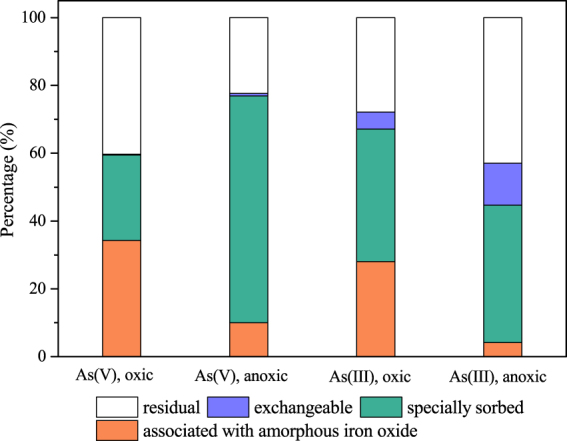
Arsenic contents and fractions in As-loaded CSF under oxic and anoxic conditions (As(V) = 100 mg/L, As(III) = 50 mg/L, CSF = 0.1 g-Fe/L, initial pH = 9.0).

The distribution of arsenic between the aqueous and solid phases was quantified to determine the different modes of binding between arsenic and CSF under oxic and anoxic conditions. The arsenic content in a suspension can be divided into five distinct fractions, including the residual arsenic in solution, exchangeable arsenic, specially sorbed arsenic, arsenic associated with amorphous iron oxides and arsenic associated with well-crystallized iron oxides. Figure 2 indicates that unlike in anoxic conditions, a large amount of As(V) and As(III) were removed through association with amorphous iron oxide under oxic conditions, with amorphous iron oxide fractions of 34.2% and 28.0%, respectively. In the presence of O_2_, the specially sorbed fraction decreased from 66.9% to 25.2% for As(V). In addition, the exchangeable fraction on the As(III)-loaded CSF decreased from 12.4% to 5.05%. These results showed that oxygen induced coprecipitation of iron and arsenic species and inhibited the surface complexation of arsenic on CSF. The adsorption of arsenic onto minerals may form both inner-sphere and outer-sphere complexes. Outer-sphere complexes are sensitive to changes in the ionic strength while inner-sphere complexes are not^28^. Thus, the effect of the ionic strength was further investigated to determine the type of surface complexes formed (Fig. S1). The removal capacity of CSF was dependent on the ionic strength only for the removal of As(III) under anoxic conditions, and the removal efficiency of As(III) decreased from 74.0% to 41.7% when the concentration of NaCl increased from 50 mM to 100 mM. This result indicated that As(III) and As(V) mainly formed inner-sphere complexes on CSF, and a portion of the As(III) also formed outer-sphere complexes with CSF under anoxic conditions. Furthermore, this mechanism confirmed that ligand exchange occurred between CSF and both As(V) and As(III) under anoxic conditions. In addition, weak binding forces, such as electrostatic interactions and van der Waals forces, also played a role in the removal of As(III). These results were consistent with those of Jönsson and Sherman^29^, who also found that As(V) formed an inner-sphere complex on ferrous carbonate under anaerobic conditions, whereas As(III) formed a weak outer-sphere complex. The formation of different surface complexes of As(V) and As(III) may be due to the differences in the surface charges of these arsenic species. At the given pH, As(III) (pKa_1_ = 2.20, pKa_2_ = 6.97, pKa_3_ = 11.53^30^) exists as uncharged H_3_AsO_3_ molecules, while As(V) (pKa_1_ = 9.22, pKa_2_ = 12.13, pKa_3_ = 13.40^30^) exists as negatively charged oxyanions. In general, oxyanions such as phosphate, chromate, and selenate are prone to form inner-sphere complexes on iron (hydr)oxides^31–34^. Similarly, As(V) was speculated to have a higher affinity than As(III) for CSF.

**Figure 3:**
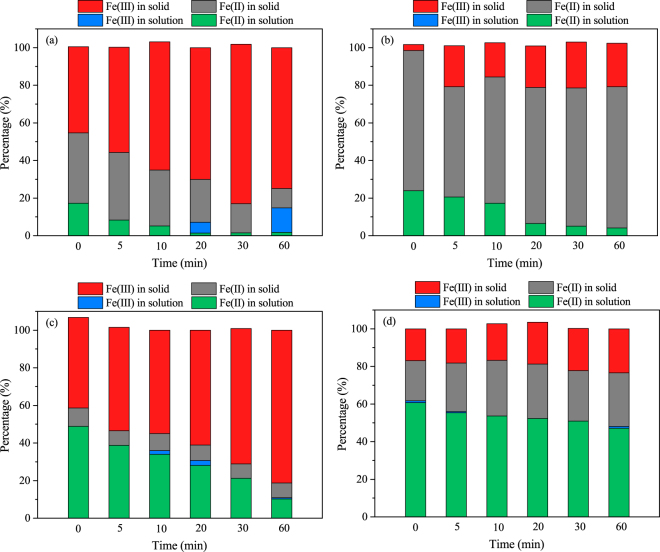
Changes in the ratios of ferric species during the removal of arsenic by CSF under oxic and anoxic conditions (As(V) = 100 mg/L, As(III) = 50 mg/L, CSF = 0.1 g-Fe/L, initial pH = 9.0); (**a**) As(V), oxic conditions; (**b**) As(V), anoxic conditions; (**c**) As(III), oxic conditions; and (**d**) As(III), anoxic conditions.

The changes in the ratios of ferric species during the reaction were further investigated. As presented in Fig. 3, in the presence of oxygen, the concentrations of Fe(II) in solution and the solid state obviously decreased with increasing reaction time, while the ratio of Fe(III) in the solid state increased significantly. This indicated that the CSF was rapidly oxidized and transformed to Fe(III) hydroxide under oxic conditions. In contrast, under anoxic conditions, the ferric species mainly existed as solid Fe(II) in the presence of As(V), and in the presence of As(III), the ratio of dissolved ferrous ions remained nearly constant. Ferrous carbonate can be oxidized by O_2_ and transform into lepidocrocite and goethite during arsenic adsorption, which can be described by equations (1) and (2)^35^:

1 $$F{\rm{e}}C{O}_{3}+{H}_{2}O\leftrightarrow F{e}^{2+}+HC{O}_{3}^{-}+O{H}^{-}$$

2 $$2F{{\rm{e}}}^{2+}+\frac{1}{2}{O}_{2}+{H}_{2}O\leftrightarrow 2F{\rm{e}}OOH+4{H}^{+}$$

During the reactions, the arsenic species were incorporated into the iron oxyhydroxides. Renard *et al*. proposed that siderite dissolves and releases Fe(II) into solutions, and the iron oxides and oxyhydroxides generated from the subsequent oxidation and precipitation of this Fe(II) then sequester arsenic^36^. In contrast, under anoxic conditions, Fe(II) oxidation is inhibited, and ferrous ions remain in solution. Thus, the contribution of dissolved ferrous ions to the removal of arsenic needs to be examined. After the addition of 0.1 g/L dissolved ferrous ions at pH 9.0, the concentration of As(III) remained nearly constant under both oxic and anoxic conditions. In contrast, 60.3% and 68.3% of As(V) was removed under oxic and anoxic conditions, respectively (Fig. S2). These results demonstrated that at a given pH and ferrous concentration, minimal precipitation of Fe(II) with As(III) can occur under anoxic conditions. However, As(V) and ferrous ions can precipitate under anoxic conditions, which provides another pathway for sequestering As(V).

### XRD characterization and FTIR analysis

**Figure 4:**
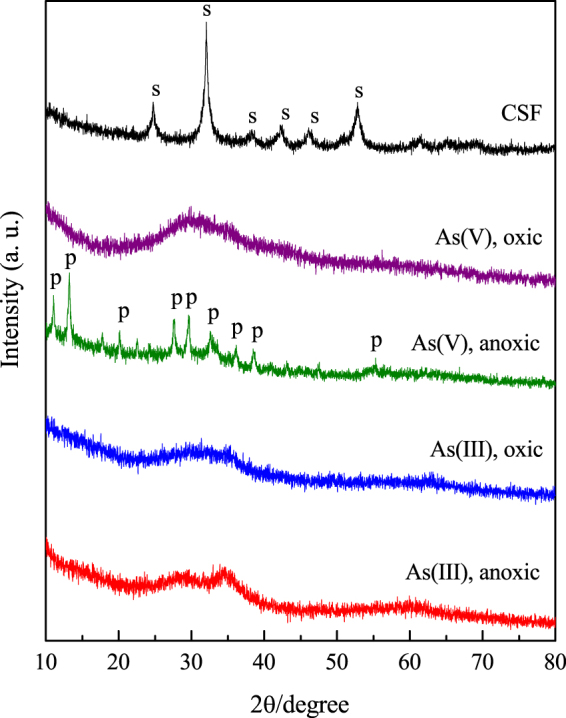
XRD patterns of pristine CSF and As-loaded CSF under oxic and anoxic conditions (As(V) = 100 mg/L, As(III) = 50 mg/L, CSF = 0.1 g-Fe/L, initial pH = 9.0). The peaks indicate siderite (s) and parasymplesite (p).

As shown in Fig. 4, the XRD pattern of solid CSF contained obvious diffraction peaks corresponding to siderite. In the presence of O_2_, the As(V)- and As(III)-loaded CSF materials both showed a broad band at 28°. Amorphous ferric arsenate (or amorphous scorodite) is known to exhibit a broad band at 28°^37^. Thus, the XRD results indicates that the As(V)-loaded CSF was amorphous ferric arsenate and the As(III)-loaded CSF was an As-Fe-containing precipitate. Under anoxic conditions, the diffraction peaks of the As(V)-loaded CSF corresponded to crystalline parasymplesite (Fe(II)_3_(AsO_4_)_2_·8H_2_O, pK_a_ = 33.25^38^). Lin *et al*.^39^ used goethite, lepidocrocite and green rust to remediate arsenic and found that green rust was also transformed to parasymplesite in a reducing environment, resulting in significant attenuation of arsenic. Parasymplesite was previously demonstrated to be a good candidate for treating arsenic-bearing solid residuals, and it also passed the toxicity characteristic leaching procedure (arsenic leaching < 5 mg/L)^40^. The removal of As(V) under anoxic conditions by CSF was thus established as a promising approach for achieving highly efficient arsenic removal and forming stable products at the same time. The As(III)-loaded CSF also showed a band at 28° under anoxic conditions. Since As(III) was determined to bind to CSF through surface complexation and because no additional precipitate was formed, this precipitate was most likely a mixture of As(III) and structural Fe(II). The FTIR spectra of the precipitates were recorded to examine the binding interactions of arsenic and iron (as shown in Fig. S3). The bands observed at 1402 cm^−1^ and 1121 cm^−1^ for pristine CSF were attributed to the characteristic asymmetric and symmetric stretching vibration bands of CO_3_ in siderite. After reaction under both oxic and anoxic conditions, the band for CO_3_ disappeared, indicating that CSF underwent a mineral transformation. Single bands at 830 cm^−1^ and 822 cm^−1^ then emerged in the As(V)-loaded CSF under oxic and anoxic conditions, and these bands were attributed to the stretching vibrations of As-O units coordinated to iron atoms, e.g., As-O-Fe^21,41^. The band observed at 816 cm^−1^ for the As(III)-loaded CSF under oxic conditions was also attributed to the vibration of As-O-Fe bonds. In addition, the redshift in this band to 799 cm^−1^ in the As(III)-loaded CSF under anoxic conditions was assigned to As-O-Fe bidentate-binuclear coordination^21^. However, the band at 861 cm^−1^ corresponding to the uncomplexed/unprotonated As-O unit was not observed.

### XPS analysis

**Figure 5:**
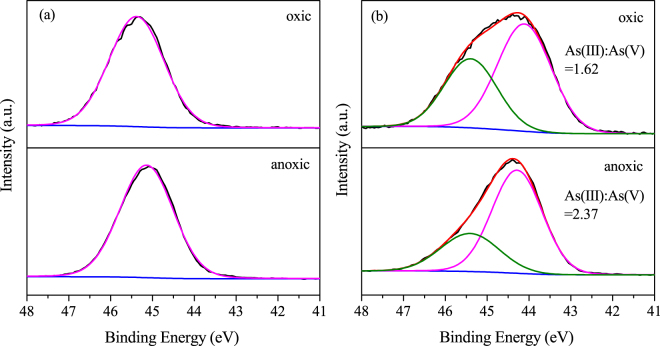
As 3d XPS spectra of (**a**) As(V)-loaded CSF and (**b**) As(III)-loaded CSF under oxic and anoxic conditions (As(V) = 100 mg/L, As(III) = 50 mg/L, CSF = 0.1 g-Fe/L, initial pH = 9.0).

The redox transformation of arsenic is crucial for the removal of arsenic species. Thus, the redox states of arsenic on the surface of the As-loaded CSF materials were analysed by XPS. As shown in Fig. 5, the spectra were separated into two curves, with As(III) and As(V) observed at binding energies of ~44.2 eV and ~45.6 eV, respectively. Only As(V) was detected on the As(V)-loaded CSF under both oxic and anoxic conditions, suggesting that reduction to As(III) did not occur on the CSF. Likewise, the reduction of As(V) by reactive Fe(II), i.e., siderite and green rust, has not been reported previously^26,29^. In addition, the peak area ratios of As(III)/As(V) on the surface of the As(III)-loaded CSF were 1.62/1 and 2.37/1 under oxic and anoxic conditions, respectively. This result suggested that As(III) was partially oxidized to As(V) under oxic and anoxic condition, and more As(III) was oxidized in the presence of O_2_. Previous studies confirmed that As(III) can be oxidized by reactive oxygen species such as ·OH as well as the Fe(IV) species that are generated from Fenton-like reactions in the presence of O_2_. Recently, ·OH was confirmed to be produced from the oxygenation of siderite under circumneutral conditions^42^. This reaction may have contributed to the oxidation of As(III). Under anoxic conditions, traces of oxygen resulted in limited partial oxidation of CSF. Furthermore, the coexistence of iron oxyhydroxides and aqueous Fe(II) has been reported to induce rapid oxidation of As(III) to As(V) under anoxic conditions^43,44^. The authors of those studies proposed that the reactive intermediate Fe(III) phase promoted electron transfer and subsequent As(III) oxidation. This process may explain the observed oxidation of As(III) under anoxic conditions based on the presence of both ferrous ions and Fe(II)/Fe(III) minerals. The identical redox changes of As(V) in the presence and absence of O_2_ indicated that the valence change of the arsenic species was not responsible for the different sorption densities determined for As(V). In contrast, higher oxidation efficiency of As(III) was one of the reasons for higher sorption density of As(III) under oxic condition than under anoxic condition.

### Effect of As/Fe molar ratio

**Figure 6:**
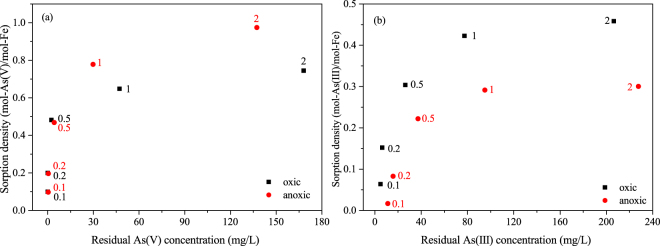
Relationship between the sorption density and the residual arsenic concentration on the removal of (**a**) As(V) and (**b**) As(III) by CSF under oxic and anoxic conditions (CSF = 0.1 g-Fe/L, As/Fe molar ratio = 0.1–2, initial pH = 7.0. The data label is the initial As/Fe molar ratio).

Since the As/Fe molar ratio is an important factor that may affect the removal capacity and mechanism of CSF, the effect of As/Fe was further investigated. Rich in bicarbonate ions, CSF has a good buffering capacity. Due to the buffering capacity of CSF (pH_zpc_ = 6.23), the pH value dropped to 6–8 (Fig. S4) after reaction started. However, at a high As/Fe ratio, the buffering capacity would be weaker and the pH value remains steady during the reaction, which in turn affects the sorption density. To avoid this problem, a neutral pH (7.0) was used in the sorption experiments. As shown in Fig. 6, at pH 7.0, the sorption density of As(V) was higher under anoxic conditions than oxic conditions for As/Fe ratios from 0.1 to 2. In contrast, As(III) showed an obviously higher density on CSF under oxic conditions over the same range of As/Fe ratios. This result indicated that oxygen exerted opposite effects on the removal of As(III) and As(V) over a wide range of As/Fe ratios.

**Figure 7:**
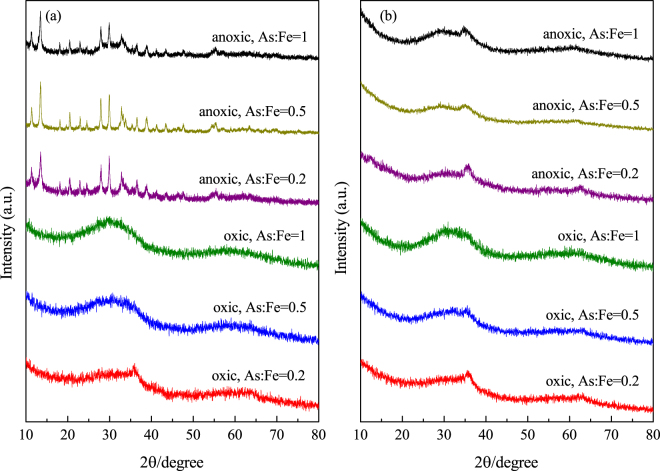
XRD patterns of (**a**) As(V)-loaded CSF and (**b**) As(III)-loaded CSF under oxic and anoxic conditions (CSF = 0.1 g-Fe/L, As/Fe molar ratio = 0.2–1, initial pH = 7.0).

The removal mechanism of arsenic under various As/Fe ratio was further investigated by XRD analysis. The XRD spectrum of poorly crystalline ferric arsenate shows two broad XRD bands at 28° and 58°, while the spectrum of two-line ferrihydrite shows two bands at 34° and 61°^20^. As shown in Fig. 7, under oxic condition, when As(III)-loaded CSF and As(V)-loaded CSF were generated at an As/Fe ratio of 0.2, the XRD patterns corresponded to a two-line ferrihydrite. As As/Fe ratio increased to 0.5 and 1, the bands shifted to the bands of poorly crystalline ferric arsenate and Fe-As-containing precipitates, respectively. This indicated that precipitation was dominated in the coprecipitation process under oxic condition as As/Fe increased. Many researchers have found that surface precipitation was involved along with increase of the molar ratio of ions and adsorbents^45,46^. In contrast, under anoxic condition, the XRD patterns of As(V)-loaded CSF corresponded to parasymplesite and As(III)-loaded CSF was attributed to a mixture of As(III) and structural Fe(II) for As/Fe ratios from 0.2 to 1. This demonstrated that the mechanism under anoxic condition remained the same when As/Fe was from 0.2 to 1.

### Mechanisms of As-containing precipitate formation

**Figure 8:**
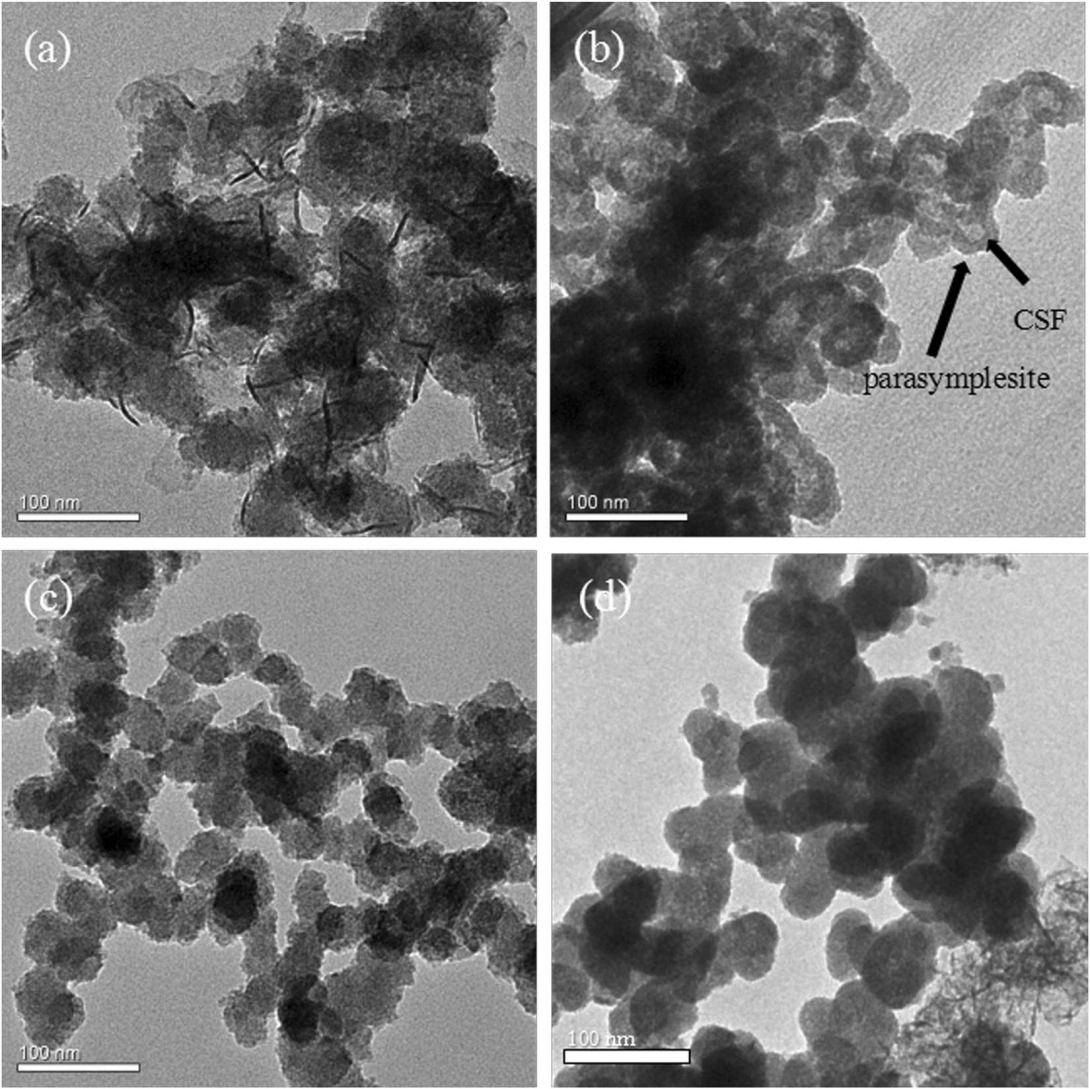
TEM images of the As-loaded CSF materials under oxic and anoxic conditions (As(V) = As(III) = 20 mg/L, CSF = 0.05 g-Fe/L, initial pH = 7.0); (**a**) As(V), oxic conditions; (**b**) As(V), anoxic conditions; (**c**) As(III), oxic conditions; and (**d**) As(III), anoxic conditions.

To determine the mechanism of precipitate formation, a lower initial arsenic concentration was used for TEM characterization of the As-loaded CSF. As depicted in Fig. 8, under oxic conditions, the As(V)- and As(III)-loaded CSF materials consisted of uniform spherical particles with an average diameter below 100 nm. The substantial presence of arsenic enhanced the As-Fe interactions and hindered the normal Fe-Fe crystallization by oxygen, thus restricting the growth of crystals. The small particle size was consistent with the amorphous peak shapes observed in the XRD patterns. Under anoxic conditions, the As(V)-loaded CSF consisted of particles coated with a layer. Under anoxic condition, CSF could be protected from mineral oxidation and transformation. Also, the coexistence of ferrous ions and arsenate enable the surface precipitation of parasymplesite on CSF. The particle inside the layer was speculated to be pristine CSF, and the coating consisted of surface precipitates of parasymplesite. Surface precipitation was previously found to occur during the adsorption of As(V) on goethite^47^ and ferrihydrite^21^. The general process involved the dissolution of Fe-based minerals, ternary complexation of Fe(III) and subsequent precipitation of As(V). Similarly, the process of parasymplesite formation in the current system was speculated to proceed as follows: As(V) first adsorbed onto CSF through surface complexation, the dissolved Fe(II) then formed a ternary complex, and finally, Fe(II) and As(V) formed precipitates on the surface of the CSF. The absence of layered structure under oxic condition may be because CSF underwent mineral oxidation and transformation in the presence of O_2_. The original structure of CSF was broken and arsenic was incorporated accompanied by the generation of iron oxyhydroxides. The structure and morphology of the As-loaded CSF materials were also analysed by SEM at a high arsenic loading. As shown in Fig. S5, under anoxic conditions, the As(V)-loaded CSF consisted of flaky particles that corresponded to crystalline parasymplesite. In contrast, all the other precipitates consisted of uniform spherical particles that were consistent with those observed in the TEM images. According to Table 1, the As/Fe wt% in the As(V)-loaded CSF was higher under anoxic conditions than under oxic conditions, while that in the As(III)-loaded CSF was higher under oxic conditions than under anoxic conditions. This suggested that more As(V) ions were incorporated in CSF under anoxic conditions, whereas more As(III) ions were incorporated under oxic conditions, which was consistent with the results of the batch experiments.

**Table 1:** Energy-dispersive X-ray analyses of the As-loaded CSF materials under oxic and anoxic conditions.

Contents	CSF + As(V), oxic	CSF + As(V), anoxic	CSF + As(III), oxic	CSF + As(III), anoxic
	Weight%	Weight%	Weight%	Weight%
Fe	27.92	24.04	15.19	30.77
As	10.05	16.72	6.96	12.64
As/Fe	0.360	0.696	0.458	0.411

(As(V) = As(III) = 20 mg/L, CSF = 0.05 g-Fe/L, initial pH = 7.0).

**Figure 9:**
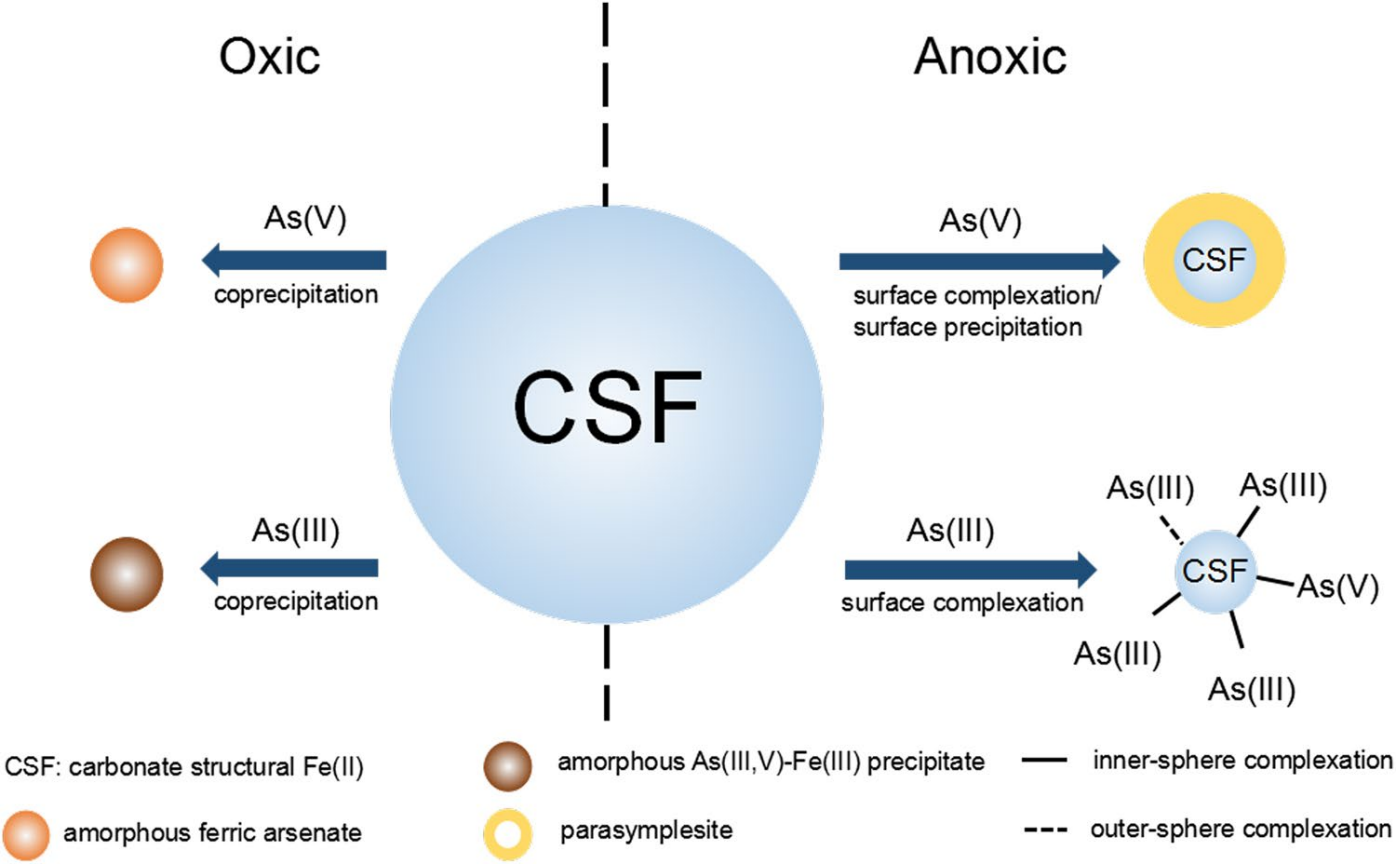
Schematic representation of arsenic removal by CSF under oxic and anoxic conditions.

The proposed mechanism of arsenic removal by CSF under oxic and anoxic conditions is shown in Fig. 9. Under anoxic conditions, CSF had a high affinity for negatively charged As(V) and thus formed an inner-sphere surface complex. The surface-bound As(V) on the CSF and the dissolved ferrous ions formed surface precipitates and produced parasymplesite. As(III), which is neutral at the given pH, had a weaker affinity for CSF and was unable to form a precipitate with ferrous ions. Therefore, the removal of As(III) occurred mainly through outer- and inner-sphere complexation, which showed a poor removal efficiency under anoxic conditions. In the presence of oxygen, CSF underwent mineral transformations, and the dissolved Fe(II) species hydrolysed and precipitated to form Fe(III) (oxy)hydroxides. During this process, both As(III) and As(V) were removed through coprecipitation with the iron species. Generally, coprecipitation provides a higher removal efficiency than adsorption towards metal(loid)s because the pollutants are incorporated into the adsorbents rather than simply adsorbed on the adsorbent surface. This process had a smaller removal efficiency than that of the surface complexation/precipitation of crystalline parasymplesite (Fe(II)_3_(AsO_4_)_2_·8H_2_O). This result may be due to the high Fe/As molar ratio of 3:2 in the precipitate. The reductive dissolution of Fe(III) (oxy)hydroxides has been widely accepted to release dissolved arsenic into water^48^. However, secondary Fe(II)-bearing minerals may resequester this dissolved arsenic^49^. This study sheds light on the cause of disagreement in the literature about the removal of arsenic under oxic/anoxic environments based on the type of arsenic species present. During the reduction of Fe(III) (oxy)hydroxides, As(V) may be immobilized in secondary Fe(II)-bearing minerals, whereas As(III) may be released. Future investigations should focus on creating reducing and oxidizing environments to enhance the sequestration of As(V) and As(III), respectively, by structural Fe(II).

## Conclusions

The arsenic sorption density revealed that a higher removal efficiency was obtained in the absence of O_2_ for the removal of As(V) and in the presence of O_2_ for the removal of As(III) by CSF. Dissolved oxygen promoted coprecipitation and restrained the surface complexation of arsenic with CSF. As(V) did not change valence states under both oxic and anoxic conditions. Thus minimally contributed to the observed differences in the sorption density in the presence and absence of O_2_. However, more As(III) was oxidized in the presence of O_2_. The mechanism proposed to explain the opposite effects of O_2_ was as follows: Under anoxic conditions, As(V) first bound to CSF by surface complexation, followed by surface precipitation, which promoted the removal of As(V) through the crystallization of parasymplesite. Meanwhile, the oxidation of structural Fe(II) was accompanied by a coprecipitation process, and the recombination of Fe(III) and As(III) together with more oxidation of As(III) in the presence of O_2_ resulted in a higher removal efficiency of the neutral As(III) molecules. This study suggests that CSF may be an effective material for removing As(III) and As(V) from wastewater with high arsenic content, and a high arsenic removal efficiency can be achieved in practice by controlling the O_2_ content.

## Methods

### Chemicals and materials

Stock solutions of 1000 mg/L As(III) and As(V) were prepared from NaAsO_2_ (Sigma–Aldrich) and Na_2_HAsO_4_·12H_2_O (Sigma–Aldrich), respectively. The solutions were prepared fresh for each batch experiment. Oxalic acid (purity 99%), ammonium sulfate (purity > 99%), ammonium dihydrogen phosphate (purity > 99%), sodium oxalate (purity 98%), and ascorbic acid (purity > 99.7%) were purchased from Sinopharm Chemical Reagent (Shanghai, China). CSF was synthesized from FeSO_4_·7H_2_O (Aladdin, purity > 99%) and NH_4_HCO_3_ (Aladdin, purity > 99%). The [Fe(II)]/[HCO_3_^−^] molar ratio was controlled at 1:2. FeSO_4_·7H_2_O and NH_4_HCO_3_ were dissolved and mixed in deoxygenated water on a magnetic stirrer at room temperature (22 °C). The obtained milky-white suspension was considered to be nascent CSF. CSF was freshly prepared before use, and the reaction was conducted under a high-purity nitrogen (>99.9%) atmosphere to avoid oxidation. All solutions were prepared with ultrapure water (18 MΩ·cm) from a Millipore water purification system (Bedford, USA).

### Experimental procedures

All batch experiments were carried out in 90 mL solutions under magnetic stirring at room temperature (22 °C). Under oxic conditions (DO = 8.75 mg/L), the experiments were conducted in beakers and exposed to the ambient air. Under anoxic conditions, headspace vials sealed with caps were used, and the solutions were purged with nitrogen gas for 20 min in advance to eliminate dissolved oxygen. The initial pH values of the solutions were adjusted with NaOH and HCl, and no attempt was made to maintain a constant pH during the reactions. The reactions were initiated by the addition of the CSF suspension to the solution containing As(III)/As(V). The suspension was sampled at given time intervals, filtered through a 0.22-μm membrane filter, and acidified for analysis. The sequential chemical extraction procedure described by Shao *et al*.^3^ was used to investigate the arsenic distribution in the aqueous and solid phases.

### Analytical methods

The dissolved arsenic concentrations were analysed using inductively coupled plasma optical emission spectrometry (ICP-OES, Agilent 720ES, USA). The concentrations of ferrous iron and total iron in the solution and solids were determined by the 1,10-phenanthroline colorimetric method using a UV-visible spectrophotometer at 510 nm. After filtration, the solid products were rinsed, centrifuged and freeze-dried for later characterization. The mineral composition of the pristine and used adsorbents was determined by XRD with a Bruker D8 Advance powder X-ray diffractometer (Germany) using a Cu Kα (k = 1.54178 Å) radiation source operated at 40 kV and 40 mA. The diffraction angle (2θ) was set from 10° to 80° with a scanning speed of 1°/min and a step size of 0.02°. FTIR spectra of the samples were recorded on a Nicolet 5700 spectrometer over a range of 4000–400 cm^−1^. The binding energies and atomic ratios of arsenic on the surface of the materials were analysed by X-ray photoelectron spectroscopy on a ESCALAB 250XI (Thermo Fischer, USA) equipped with a rotating Al anode generating Al Kα X-ray radiation at 1486.6 eV. Morphological analyses of the solid phases were conducted using a field emission SEM (Hitachi S-4800, Japan) and TEM (JEOL JEM 2011, Japan) with energy-dispersive X-ray analyses. The concentration of dissolved oxygen was determined by a portable meter (WTW Multi 3430, Germany).

### Data availability

The datasets generated during the current study are available from the corresponding author on reasonable request.

## Electronic supplementary material

Supplementary information
