# Supplementary material for: Opposite effects of dissolved oxygen on the removal of As(III) and As(V) by carbonate structural Fe(II)
Source: Sci Rep. 2017 Dec 5;7:17015. doi: 10.1038/s41598-017-17108-4 (PMC5717268; doi:10.1038/s41598-017-17108-4)
Supplement: Supplementary file 1 — Supplementary information [file 41598_2017_17108_MOESM1_ESM.pdf]

Supporting Information

Opposite effects of dissolved oxygen on the removal of As(III) and As(V) by carbonate structural Fe(II)

Zeyuan Tian[1], Yong Feng[2], Yiyi Guan[1], Binbin Shao[1], Yalei Zhang[1], Deli Wu[1]*

[1] State Key Laboratory of Pollution Control and Resources Reuse, College of Environmental Science & Engineering, Tongji University, Shanghai, 200092, P.R. China.

[2] Department of Civil Engineering, The University of Hong Kong, Pokfulam Road, Hong Kong.

*Correspondence and requests for materials should be addressed to W.-D.L. (email: wudeli@tongji.edu.cn).

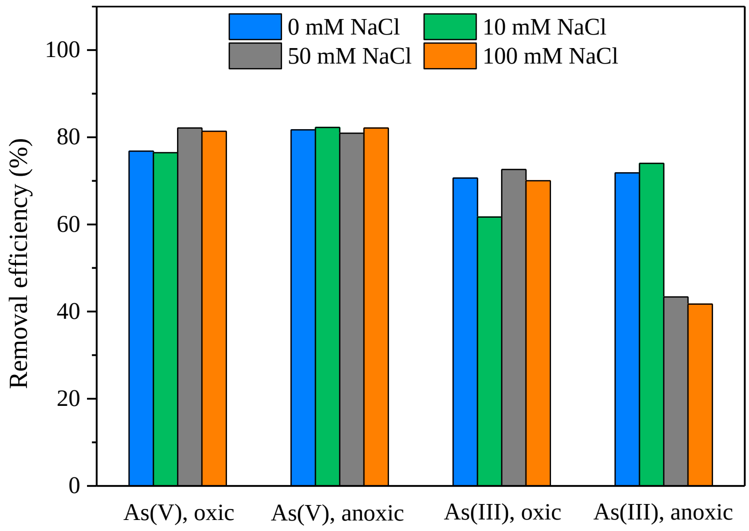

Figure S1. Effect of ionic strength on the removal of As(V) and As(III) by CSF under oxic and anoxic conditions (As(V)=25 mg/L, CSF=0. 025 g-Fe/L, As(III)=25 mg/L, CSF=0.05 g-Fe/L, initial pH=9.0).

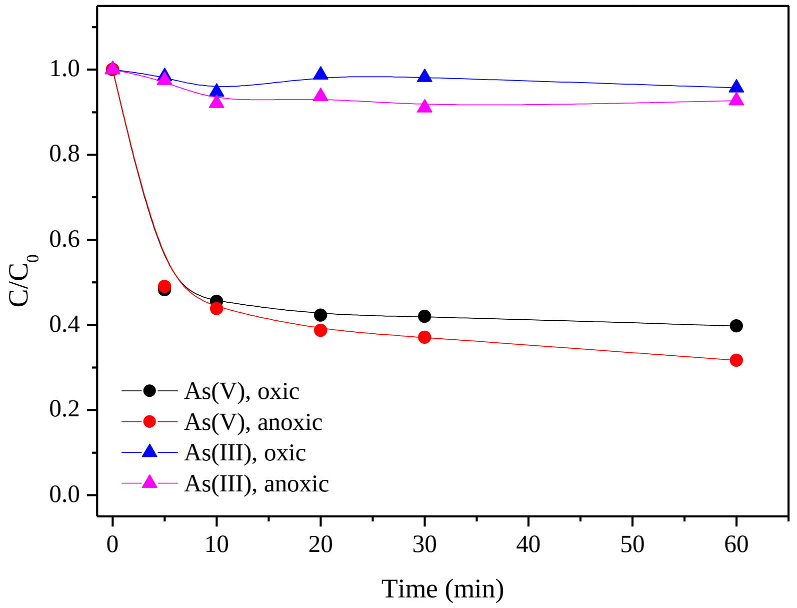

Figure S2. Effect of dissolved ferrous ions on the removal of As(V) and As(III) by CSF under oxic and anoxic conditions (As(V)=100 mg/L, As(III)=50 mg/L, Fe(II)=0.1 g/L, initial pH=9.0).

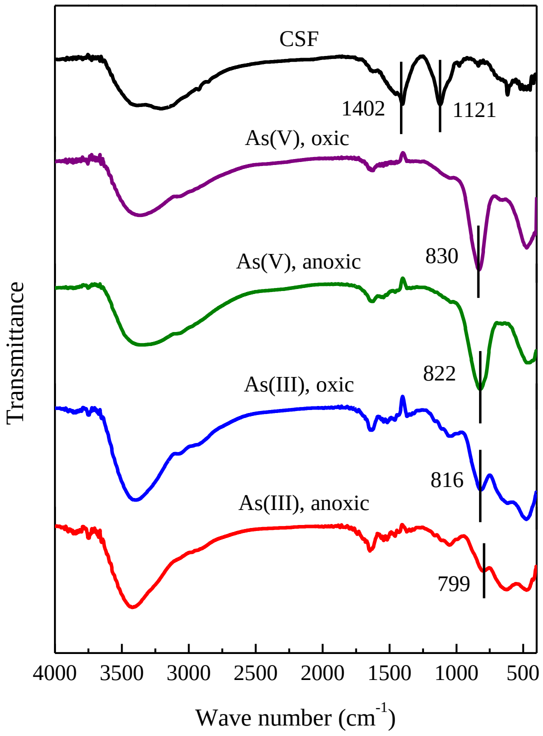

Figure S3. The FT-IR spectra of pristine CSF and As-loaded CSF under oxic and anoxic conditions (As(V) =100 mg/L, As(III)=50 mg/L, CSF=0.1 g-Fe/L, initial pH=9.0).

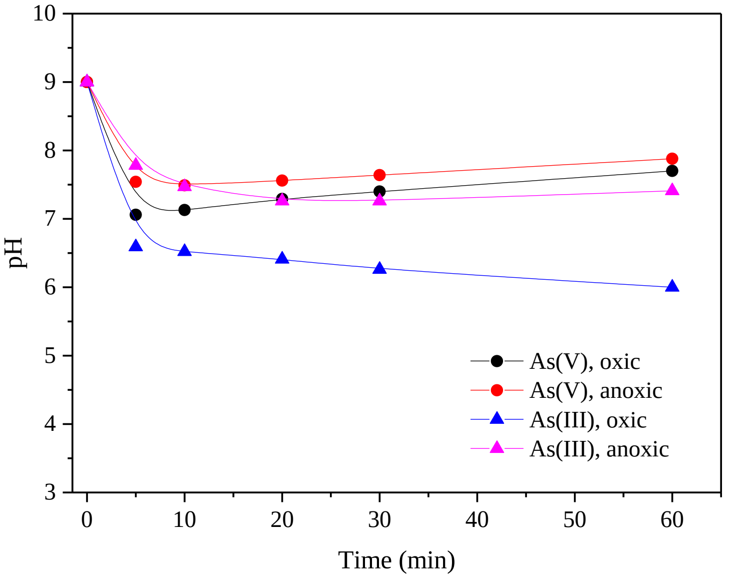

Figure S4. Changes of pH on the removal of As(V) and As(III) by CSF under oxic and anoxic conditions (As(V)=100 mg/L, As(III)=50 mg/L, CSF=0.1 g-Fe/L, initial pH=9.0).

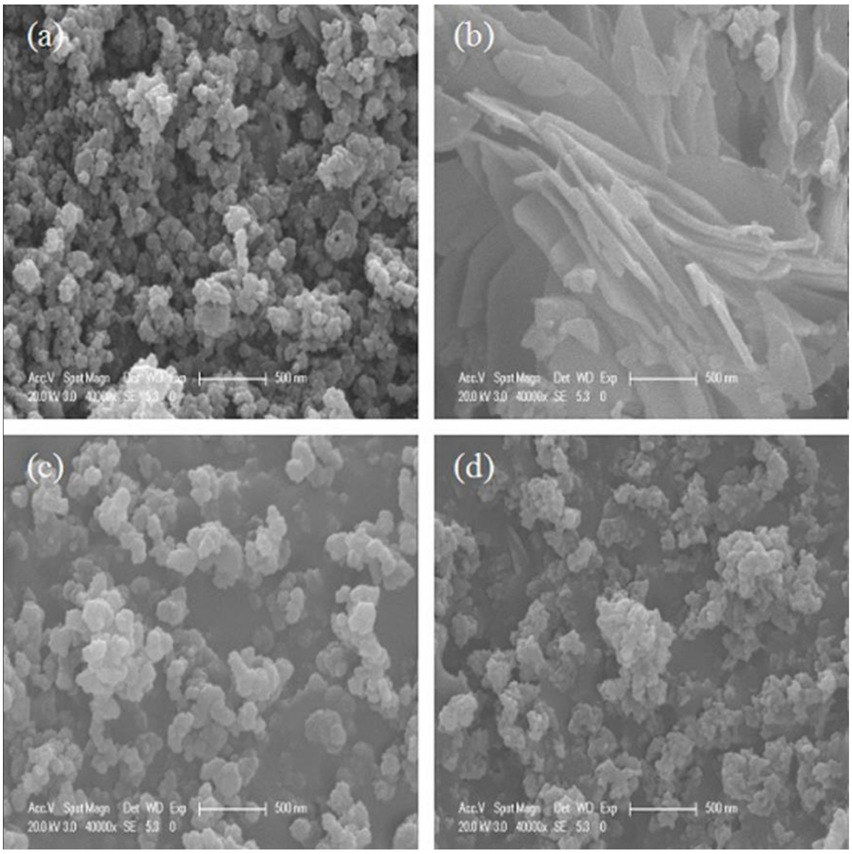

Figure S5. SEM images of As-loaded CSF under oxic and anoxic conditions (As(V)=100 mg/L, As(III)=50 mg/L, CSF=0.1 g-Fe/L, initial pH=9.0, (a) As(V), oxic, (b) As(V), anoxic, (c) As(III), oxic, (d) As(III), anoxic).
